# Case-Control Study on Risk Factors of CRE Infection in the ICU

**DOI:** 10.2174/0118715303410602251128055707

**Published:** 2026-02-04

**Authors:** Li Wang, Jing Xu, Lianxiang Li, Fangliang Lei

**Affiliations:** 1 Office of Hospital Infection Management, Shaanxi Provincial People’s Hospital, Xi’an, Shaanxi, 710068, China

**Keywords:** Intensive care unit, carbapenem-resistant *Enterobacteriaceae* (CRE), risk factor, unmatched case-control study, broad-spectrum penicillins, *Escherichia coli*

## Abstract

**Introduction:**

In recent years, the increasing prevalence of carbapenem-resistant *Enterobacteriaceae* (CRE) poses a serious threat to clinical anti-infection therapy. This study aims to identify risk factors for CRE infection among intensive care unit (ICU) patients.

**Methodology:**

An unmatched case-control study based on a hospital was performed. Data on the general condition of patients, disease scores, invasive procedures, and the use of antibiotics before infection were collected. Univariate and multivariate Logistic regression were performed to analyze the risk factors for CRE infection.

**Results and Discussion:**

The univariate analysis results indicated that higher APACHE-II scores, tracheal intubation, urinary catheterization, gastric tube insertion, prior use of antibiotics, use of three or more types of antibiotics, and the use of carbapenems, broad-spectrum penicillins, antifungals, and aminoglycosides were potential risk factors for CRE infection in ICU patients. In contrast, a higher GCS score appeared to be a potential protective factor against CRE infection in ICU patients. Multivariate Logistic regression analysis showed that higher APACHE-II score (OR=2.425, 95% CI:1.713~3.946), tracheal intubation (OR=3.459, 95% CI:2.617~7.148), and the use of antibiotics before infection (OR=2.704, 95% CI: 2.013~5.358) were the risk factors of CRE infection in ICU patients; higher GCS score (OR=0.459, 95% CI: 0.349~0.725) was the protective factors of CRE infection in ICU patients.

**Conclusion:**

Multiple risk factors for CRE infections in ICU patients are closely related to patients' clinical conditions and treatment regimens. Clinically, it is essential to regularly screen ICU patients with high-risk factors for CRE infections, enhance antimicrobial stewardship, perform pathogen cultures and identification, and minimize invasive procedures. These measures are critical for preventing and reducing CRE infections.

## 1 INTRODUCTION

Carbapenem-resistant *Enterobacteriaceae* (CRE) are bacteria that exhibit resistance to carbapenem antibiotics (including imipenem, meropenem, ertapenem, doripenem, *etc*.) or carry carbapenem-resistant enzymes [1]. Clinically, carbapenem-resistant *Klebsiella pneumoniae* (CRKP) and *Escherichia coli* are the most commonly observed. The first case of CRKP was reported in the United States in 2001 [2], and since then, CRE has rapidly spread worldwide. In a global multicenter study, Sader *et al.* [3] collected 8,787 *Enterobacteriaceae* isolates from 64 medical centers worldwide and reported an overall CRE resistance rate of 4.5% in 2019. According to WHO GLASS data in 2021 [4], the resistance rates of meropenem and imipenem for CRKP were 12.34% and 10.63%, respectively, while for carbapenem-resistant *Escherichia coli*, the meropenem resistance rate was 0.9% and the imipenem resistance rate was 1.3%.

China is among the countries with a significant prevalence of CRE; data from the China Antimicrobial Resistance Surveillance System (CHINET) indicate a yearly increase in CRE detection rates. From 2005 to 2022, the resistance rate of *Klebsiella pneumoniae* to imipenem increased from 3.0% to 22.6%, and its resistance to meropenem rose from 2.9% to 24.2%. The misuse and overuse of antibiotics have made CRE infections more difficult to treat, prolonging ICU stays and increasing mortality risk. Moreover, CRE exhibits resistance to most conventional antibiotics, presenting significant challenges to clinical management [5].

Therefore, both basic and clinical studies are necessary to develop effective antibacterial agents, while proactive measures should be implemented to control the prevalence and spread of CRE. Patients in intensive care units (ICUs) often have critical conditions, compromised immunity, and frequent invasive procedures, making them high-risk populations for CRE infections [6]. Currently, effective treatment options for CRE infections remain limited [7]. Early proactive screening is crucial for preventing infections and improving outcomes. This study focuses on ICU patients with CRE infections and conducts a retrospective analysis of infection characteristics and risk factors to identify key determinants for controlling CRE infections. It aims to provide a reference for the prophylaxis and therapy of CRE infections.

## 2 AIMS AND OBJECTIVES OF THE STUDY

This study focuses on ICU patients with CRE infections and conducts a retrospective analysis of infection characteristics and risk factors to identify key determinants for controlling CRE infections. It aims to provide a reference for the prophylaxis and therapy of CRE infections and support clinical decision-making in antimicrobial stewardship.

## 3 METHODOLOGY

### 3.1 Setting and Sample

The research hospital is a Grade-A tertiary hospital directly under the provincial government. An unmatched case- control study was conducted in this tertiary hospital in Xi’an. A total of 498 patients admitted to the ICU from January 2020 to December 2021, with confirmed *Enterobacteriaceae* infections, were included in the study. Based on their resistance to carbapenem antibiotics, patients were classified into the CRE group (162 cases, case group) and the non-CRE group (336 controls, control group). Bacterial identification and antimicrobial susceptibility testing were performed according to the National Clinical Laboratory Procedures guidelines [8].

Inclusion criteria were patients diagnosed with hospital-acquired infections due to *Enterobacteriaceae* according to the Ministry of Health's Hospital Infection Diagnosis Standards (Trial) [9].

Exclusion criteria included (1) Patients with colonization, suspected contamination, or infections occurring within 48 hours of admission; (2) Patients with incomplete clinical data or unclear antimicrobial usage.

### 3.2 Sample Size Calculations

The proportion of patients undergoing invasive procedures in the ICU ranges from 20% to 90% [10]. Assuming that the estimated percentages of patients undergoing invasive procedures are 55.0% in cases and 39.0% in controls, with a correlation of exposure between cases and controls of zero, a type I error rate of 0.05, and a test power of 80%, the required sample sizes were calculated as 150 cases and 300 controls. Considering a potential 10% nonresponse rate, the final analysis included 162 cases and 336 controls who completed the questionnaires, which met the sample size requirement.

### 3.3 Pathogen Culture

Following the National Clinical Laboratory Operational Procedures, specimens were subjected to pathogen culture in strict accordance with the protocols [8]. Samples were inoculated on blood agar and MacConkey agar and incubated at 35°C for 18–24 hours. Bacterial identification and antimicrobial susceptibility testing were performed using the Vitek 2 Compact system. Antibiotic susceptibility was interpreted according to the United States Clinical and Laboratory Standards Institute (CLSI) 2018 guidelines [11]. *Enterobacteriaceae* were classified as CRE if the minimum inhibitory concentration (MIC) was ≥4 mg/L for imipenem or meropenem, or ≥2 mg/L for ertapenem.

### 3.4 Data Collection Tools and Methods

Information was searched and gathered using the hospital information system and the hospital infection surveillance system, including the following details: (1) demographic information: age and gender; (2) pathogen culture data: specimen source and pathogen species; (3) underlying diseases: including diabetes, cardiovascular diseases, kidney diseases, liver and biliary diseases, and respiratory diseases; (4) invasive procedures: including mechanical ventilation, retaining urinary catheters, and vascular catheters; (5) prior use of antimicrobial agents; (6) scoring assessments included the Glasgow Coma Scale (GCS) and the Acute Physiology and Chronic Health Evaluation II (APACHE-II).

### 3.5 Data Analysis

Numeric data were described using mean ± standard deviation. Classified and ordinal data were presented using frequencies and percentages. Univariate analysis (χ^2^ test) and multivariate unconditional stepwise Logistic regression were performed using IBM SPSS Statistics 23.0. Factors that were statistically significant in the univariate analysis, as well as those identified as potentially relevant in the literature, were enrolled in the multivariate model for further analysis. A *P*-value of < 0.05 was considered statistically significant. The assignment of relevant variables is shown in Table **1**.

## 4 RESULTS

### 4.1 Basic Characteristics of the Study Patients

In the CRE group, there were 162 cases, including 95 males and 67 females, with 64 patients aged <50 years and 98 patients aged ≥50 years. In the non-CRE group, there were 336 cases, comprising 195 males and 141 females, with 134 patients aged <50 years and 202 patients aged ≥50 years. There was no difference in gender and age distribution between the two groups (*P* > 0.05).

### 4.2 Distribution of CRE Infection Specimens and Pathogens

A total of 162 specimens were collected from patients in the CRE group, including 79 sputum samples (48.77%), 35 blood samples (21.60%), 31 urine samples (19.14%), 10 pleural effusions (6.17%), and 7 other specimens (4.32%). Among the 162 CRE isolates identified, there were 93 strains of *Klebsiella pneumoniae* (57.41%), 35 strains of *Enterobacter cloacae* (23.46%), 31 strains of *Escherichia coli* (20.99%), and 3 strains of other bacteria (1.85%). The details are presented in Table **2**.

### 4.3 Single-factor Analysis of Influencing Factors for CRE Infection in ICU Patients

#### 4.3.1 The Situation of Health Scores of Patients between the CRE Group and the Non-CRE Group

The APACHE-II and GCS score levels of patients in both the CRE and non-CRE groups were statistically significant, indicating that these scores are associated with CRE infections (Table **3**).

#### 4.3.2 Underlying Health Conditions of Patients between the CRE Group and the Non-CRE Group

No statistical difference was observed between the CRE and non-CRE groups regarding underlying health conditions (Table **4**).

#### 4.3.3 Invasive Procedures of Patients between the CRE Group and the Non-CRE Group

Significant differences were found between the CRE and non-CRE groups in terms of tracheal intubation, urinary catheterization, and gastric tube insertion. Compared to the non-CRE group, the CRE group had a higher proportion of patients undergoing these invasive procedures (Table **5**).

#### 4.3.4 Pre-infection use of Antibiotics between the CRE Group and the Non-CRE Group

Statistically significant differences were observed between the CRE and non-CRE groups regarding pre-infection antibiotic use, the use of three or more types of antibiotics, and the use of carbapenems, broad-spectrum penicillins, antifungals, and aminoglycosides. Compared with the non-CRE group, a higher proportion of patients in the CRE group had received antibiotics prior to infection, used three or more types of antibiotics, and were treated with carbapenems, broad-spectrum penicillins, antifungals, and aminoglycosides (Table **6**).

### 4.4 Multivariate Analysis of Influencing Factors for CRE Infections in ICU Patients

A multivariate regression model was performed. The variable indicating whether the isolated strain was CRE-positive or not was the dependent variable. Moreover, the variables that were statistically significant in the univariate analysis (APACHE-II score level, GCS score level, tracheal intubation, urinary catheterization, gastric tube insertion, pre-infection antibiotic use, use of three or more types of antibiotics, and the use of carbapenems, broad-spectrum penicillins, antifungals, and aminoglycosides) were independent variables. The results indicated that higher APACHE II scores and endotracheal intubation were risk factors for CRE infection, whereas higher GCS scores were protective factors (Table **7**).

## 5 DISCUSSION

In recent years, the prevalence of CRE transmission worldwide has increased. The high worldwide mortality rate from CRE is also one of the reasons its epidemiological monitoring is of great concern. In a study on 991 patients with CRKP collected from 71 hospitals worldwide during 2017-2018, the unadjusted 30-day mortality rate for patients with CRKP bacteremia was 34% [12]. CRE causes sporadic outbreaks and epidemics in most regions and countries worldwide, making it a focal point for the global anti-infection community [13, 14].

This implies that *Klebsiella pneumoniae* is the predominant pathogen (accounting for 57.41%) among carbapenem-resistant *Enterobacteriaceae* causing hospital infections. It is consistent with findings from several tertiary hospitals in China [15-17]. In this study, CRE-related hospital infections primarily involved the respiratory system (48.77%), followed by bloodstream infections (21.60%) and urinary tract infections (19.14%). These findings align with Liu's conclusions [18] but differ from those reported by Zhou, potentially due to variations in regional climate, hospital scale, clinical treatment practices, and patient population characteristics.

Further analysis indicated that higher APACHE II scores (OR = 2.425, 95% CI: 1.713–3.946), endotracheal intubation (OR = 3.459, 95% CI: 2.617–7.148), and a history of prior antibiotic use (OR = 2.704, 95% CI: 2.013–5.358) were the risk factors for CRE infection. In contrast, a higher GCS score (OR = 0.459, 95% CI: 0.349–0.725) was a beneficial factor against CRE infection. The underlying reason for these findings is that patients in the ICU often exhibit unstable clinical symptoms and hemodynamic instability; thus, higher APACHE II scores and lower GCS scores correlate with increased infection risk. The APACHE II score is widely used for predicting mortality risk in emergency and critically ill patients. Research by Lang *et al.* demonstrated that as APACHE II scores increase, the detection rates of various drug-resistant strains and mortality rates also rise [10, 19]. Similarly, Yuan *et al.* reported that, among critically ill patients in the ICU, those in the mortality group had higher APACHE II scores than survivors [20]. The findings of this study are consistent with these previous studies. The reason patients with CRE infection are prone to coma is that their hemodynamic stability is poor; GCS serves as a functional scoring indicator for coma, with higher scores reflecting better consciousness [21]. This study similarly concludes that higher GCS scores are protective against CRE infection. Endotracheal intubation, a common invasive procedure in the ICU, is frequently performed on patients with severe underlying diseases and compromised immunity, which can damage the respiratory barrier, create pathways for bacterial colonization, exacerbate the activation of inflammatory mediators, and increase infection risk, thereby facilitating CRE colonization [22, 23]. This finding aligns with both domestic and international research [24, 25] on factors influencing CRE infection. Additionally, prior antibiotic use is another risk factor; it can eliminate drug-sensitive bacteria, allowing resistant strains to proliferate and increasing the risk of CRE infections, consistent with findings from related domestic studies [26-28].

## CONCLUSION

In summary, to address the risk factors for CRE infection in ICU patients, it is crucial to enhance antimicrobial stewardship, conduct thorough pathogen culture and identification, minimize invasive procedures, and regularly assess the need for mechanical ventilation to facilitate timely extubation. Implementing proactive screening and effective prevention strategies is essential to prevent and reduce the occurrence of CRE infections, ultimately improving patient outcomes.

## STUDY LIMITATIONS

This study has some limitations. First, this is a retrospective study, which introduces some biases. The prospective research can be carried out further. Second, the results may not be representative. The infection cases in this study were from a single tertiary hospital, and the results and conclusions need to be further confirmed by multicenter research.

## RECOMMENDATIONS OR IMPLICATIONS FOR PRACTICE AND/OR FURTHER RESEARCH

ICU patients are at high risk for CRE infection. Hospitals should routinely screen patients with the identified high- risk factors, reasonably use broad-spectrum antibiotics judiciously, and minimize invasive procedures to reduce the incidence of nosocomial CRE infections. Furthermore, future research should investigate the molecular mechanisms of CRE transmission, evaluate the effectiveness of targeted interventions, and assess the cost-effectiveness of prevention strategies.

## Figures and Tables

**Table 1:** Variable assignment table.

Variable	Assignment
Group Status	CRE group=1, non-CRE=0
APACHEIIScore	<10 points=1, 10-20 points=2, >20 points=3
GCS Score	3-8points=1, 9-12 points=2, 13-14 points=3, >14 points=4
Underlying Diseases	None=0, Present=1
Invasive Procedures	None=0, Present=1
Prior Antibiotic Use	Not used=0, Used=1

**Table 2:** Distribution of CRE infection specimens and pathogens.

CRE Specimen Type	Quantity
Number of Patients in CRE Group n	162
CRE Detected Specimens n (%)	-
Sputum	79(48.77)
Blood	35(21.60)
Urine	31(19.14)
Pleural Effusion	10(6.17)
Skin and Soft Tissue	6(3.70)
Other	1(0.62)
CRE Pathogen Type n (%)	-
*Klebsiella pneumoniae*	93(57.41)
*Enterobacter cloacae*	35(23.46)
*Escherichia coli*	31(20.99)
*Citrobacter freundii*	2(1.23)
*Kluyvera citrophila*	1(0.62)

**Table 3:** Comparison of health scores between the CRE group and the non-CRE group.

Health Score Indicator	CRE (n=162)	Non-CRE (n=336)	χ^2^	*P*
	**n (%)**	**n (%)**		
APACHE-II Score	-	-	-	-
<10	10(6.17)	44(13.10)	27.488	**<0.001**
10~20	58(35.80)	179(53.27)		
>20	94(58.03)	113(33.63)		
GCS Score	-	-	-	-
3~8	20(12.35)	10(2.98)	26.229	**<0.001**
9~12	81(50.00)	197(58.63)		
13~14	19(11.73)	70(20.83)		
>14	42(25.92)	59(17.56)		

**Table 4:** Comparison of underlying health conditions between the CRE group and the non-CRE group.

Underlying Disease Type	CRE (n=162)	Non-CRE (n=336)	χ^2^	*P*
	**n (%)**	**n (%)**		
Cardiovascular Disease	87(53.70)	208(61.90)	3.044	0.081
Pulmonary Disease	51(31.49)	74(22.03)	3.317	0.069
Malignant Tumor	15(9.26)	51(15.18)	3.502	0.061
Liver Disease	3(1.85)	16(4.76)	2.522	0.112
Kidney Disease	10(6.14)	14(4.17)	0.959	0.327

**Table 5:** Comparison of invasive procedures between the CRE group and the non-CRE group.

Invasive Procedure	CRE (n=162)	Non-CRE (n=336)	χ^2^	*P*
	**n (%)**	**n (%)**		
Venous Catheterization	141(87.04)	281(83.63)	0.981	0.322
**Tracheal Intubation**	**122 (75.31)**	**205 (61.01)**	**9.909**	**0.002**
**Urinary Catheterization**	**86 (53.09)**	**135 (40.18)**	**7.378**	**0.007**
Surgery	79 (48.77)	183 (54.46)	1.424	0.233
Perioperative Drainage	78 (48.15)	161 (47.92)	0.002	0.961
**Gastrostomy Tube**	**61 (37.65)**	**59 (17.56)**	**24.131**	**<0.001**
Tracheostomy	27 (16.67)	41 (12.20)	2.787	0.095
Dialysis Catheterization	5 (3.09)	4 (1.19)	2.214	0.137

**Table 6:** Comparison of pre-infection antibiotic use between the CRE group and the non-CRE group.

Pre-Infection Antimicrobial Use	CRE (n=162)	Non-CRE (n=336)	χ^2^	*P*
	**n (%)**	**n (%)**		
**Pre-Infection Antimicrobial Use**	**154** **(95.06)**	**247** **(73.51)**	**32.363**	**<0.001**
**Use of ≥3 Antimicrobial Types**	**128** **(79.01)**	**146** **(43.45)**	**55.848**	**<0.001**
Cephalosporins	125 (77.16)	234 (69.64)	3.070	0.080
**Carbapenems**	**86 (53.09)**	**63 (18.75)**	**61.459**	**<0.001**
**Broad-Spectrum Penicillins**	**62 (38.27)**	**50 (14.88)**	**34.305**	**<0.001**
**Fungal Agents**	**59 (36.42)**	**42 (12.50)**	**38.680**	**<0.001**
Vancomycin	41(25.31)	66 (19.64)	2.080	0.149
**Aminoglycosides**	**30 (18.52)**	**26 (7.74)**	**12.728**	**<0.001**
Macrolides	13 (8.02)	19 (5.65)	1.021	0.312

**Table 7:** Multivariate analysis of CRE infections in ICU patients.

Variable	*β*	S.E	Wald	*P*	OR (95% CI)
**APACHE-II Score**	**0.886**	**0.273**	**10.533**	**<0.001**	**2.425 (1.713~3.946)**
**GCS Score**	**-0.779**	**0.175**	**18.940**	**<0.001**	**0.459 (0.349~0.725)**
Urethral Catheterization	0.172	0.305	0.318	0.527	1.188 (0.551~1.946)
**Tracheal Intubation**	**1.241**	**0.391**	**10.074**	**0.037**	**3.459 (2.617~7.148)**
Gastric Tube Insertion	0.241	0.396	0.370	0.074	1.723 (1.262~3.035)
**Pre-Infection Antimicrobial Use**	**0.533**	**0.184**	**8.391**	**0.005**	**2.704 (2.013~5.358)**
Use of ≥3 Antimicrobial Types	0.118	1.028	0.013	0.391	1.125 (0.584~2.701)
Carbapenems	0.717	0.758	0.895	0.192	2.048 (0.739~5.811)
Broad-Spectrum Penicillins	0.411	0.635	0.419	0.703	1.508 (0.339~4.687)
Fungal Agents	0.194	0.616	0.099	0.503	1.214 (0.528~9.36)
Aminoglycosides	0.346	0.519	0.444	0.162	1.413 (0.815~3.295)

## Data Availability

The data collection was conducted by the research team, and the data were retained by the project group. Requests to access the datasets should be directed to the corresponding author.

## LIST OF ABBREVIATIONS

CRE: Carbapenem-resistant *Enterobacteriaceae*
ICU: Intensive Care Unit
CRKP: Carbapenem-resistant *Klebsiella pneumoniae*
CHINET: China Antimicrobial Resistance Surveillance System
MIC: Minimum Inhibitory Concentration
GCS: Glasgow Coma Scale

## STANDARDS OF REPORTING

STROBE guidelines were followed.
